# Radiographic patterns and severity scoring of COVID-19 pneumonia in children: a retrospective study

**DOI:** 10.1186/s12880-023-01154-8

**Published:** 2023-11-30

**Authors:** Jumlong Saelim, Supika Kritsaneepaiboon, Vorawan Charoonratana, Puttichart Khantee

**Affiliations:** 1 Department of Radiology, Faculty of Medicine, Prince of Songkla University, Hat Yai, 90110 Thailand; 2 Department of Radiology, Hatyai Hospital, Hat Yai, 90110 Thailand; 3 Division of Infectious Diseases, Department of Pediatrics, Faculty of Medicine, Prince of Songkla University, Hat Yai, 90110 Thailand

**Keywords:** Children, Coronavirus disease, COVID-19 pneumonia, Chest radiography, Severity score

## Abstract

**Background:**

Chest radiography (CXR) is an adjunct tool in treatment planning and monitoring of the disease course of COVID-19 pneumonia. The purpose of the study was to describe the radiographic patterns and severity scores of abnormal CXR findings in children diagnosed with COVID-19 pneumonia.

**Methods:**

This retrospective study included children with confirmed COVID-19 by reverse transcriptase-polymerase chain reaction test who underwent CXR at the arrival. The CXR findings were reviewed, and modified radiographic scoring was assessed.

**Results:**

The number of abnormal CXR findings was 106 of 976 (10.9%). Ground-glass opacity (GGO) was commonly found in children aged > 9 years (19/26, 73.1%), whereas peribronchial thickening was predominantly found in children aged < 5 years (25/54, 46.3%). Overall, the most common radiographic finding was peribronchial thickening (54/106, 51%). The lower lung zone (56/106, 52.8%) was the most common affected area, and there was neither peripheral nor perihilar predominance (84/106, 79.2%). Regarding the severity of COVID-19 pneumonia based on abnormal CXR findings, 81 of 106 cases (76.4%) had mild lung abnormalities. Moderate and severe lung abnormalities were found in 21 (19.8%) and 4 (3.8%) cases, respectively. While there were no significant differences in the radiographic severity scores among the various pediatric age groups, there were significant disparities in severity scores in the initial CXR and medical treatments.

**Conclusions:**

This study clarified the age distribution of radiographic features across the pediatric population. GGO was commonly found in children aged > 9 years, whereas peribronchial thickening was predominant in children aged < 5 years. The lower lung zone was the most common affected area, and the high severity lung scores required more medical treatments and oxygen support.

## Background

The World Health Organization has officially declared coronavirus disease (COVID-19) outbreak a global pandemic. According to the largest epidemiologic survey, the radiological features of COVID-19 have been mostly described in adults. Typical chest radiographic features are peripheral or subpleural ground-glass opacities (GGOs) or consolidation. The distribution is usually bilateral with more prominence in the lower lung than in the upper lung zones [1, 2]. In adults, the radiographic features vary from negative chest radiography (CXR) [3] to diffuse disease between the 1st and 3rd week and may be associated with severe acute respiratory distress syndrome (ARDS); however, cases of children are usually mild and less severe and critical (5.9%) than cases of adults (18.5%) [4–6]. The incidence of pediatric COVID-19 pneumonia with mild clinical symptoms was low, and one-fourth to one-half of those cases had abnormal CXR [7, 8]. Moreover, children (50%) have less involvement on computed tomography (CT) than adults (91.5%) [9]. To prevent unnecessary radiation exposure, CXR is the preferred imaging procedure in clinical decisions and management of children with COVID-19 pneumonia. Additional CT findings do not change management in childhood [10, 11].

Although the heterogeneous image quality of pediatric CXR affects the interpretation of radiographic findings, CXR highlights the spectrum of findings in COVID-19 [12]. A previous study showed that peribronchial thickening is a major radiographic feature in children, followed by GGOs [13]. The severity scores of CXR focuses on the assessment of COVID-19 pneumonia in adults [14], but the lung scoring system for pediatric CXR has not been widely reported [15, 16]. Therefore, this study aimed to assess the radiographic patterns and severity scores on CXR in children with confirmed COVID-19 pneumonia. We also evaluated the association between the CXR findings, lung scoring system, and clinical patterns (e.g., symptoms, medical treatment, and cycle threshold value).

## Methods

### Study population

This retrospective study was conducted in two hospitals; Songklanagarind Hospital (HOSPITAL #1) and Hatyai Hospital (HOSPITAL #2). The study was approved by the local Human Research Ethics Committee (REC. 64–564-7–3). We reviewed all CXR findings of children with confirmed COVID-19 based on positive reverse transcriptase-polymerase chain reaction (RT-PCR) for severe acute respiratory syndrome-corona virus-2 (SARS-CoV-2) from nasopharyngeal swabs, who were admitted to the hospitals, between April 1^st^, and December 31^st^, 2021.

### Sample size calculation and sampling technique

**Fig. 1:**
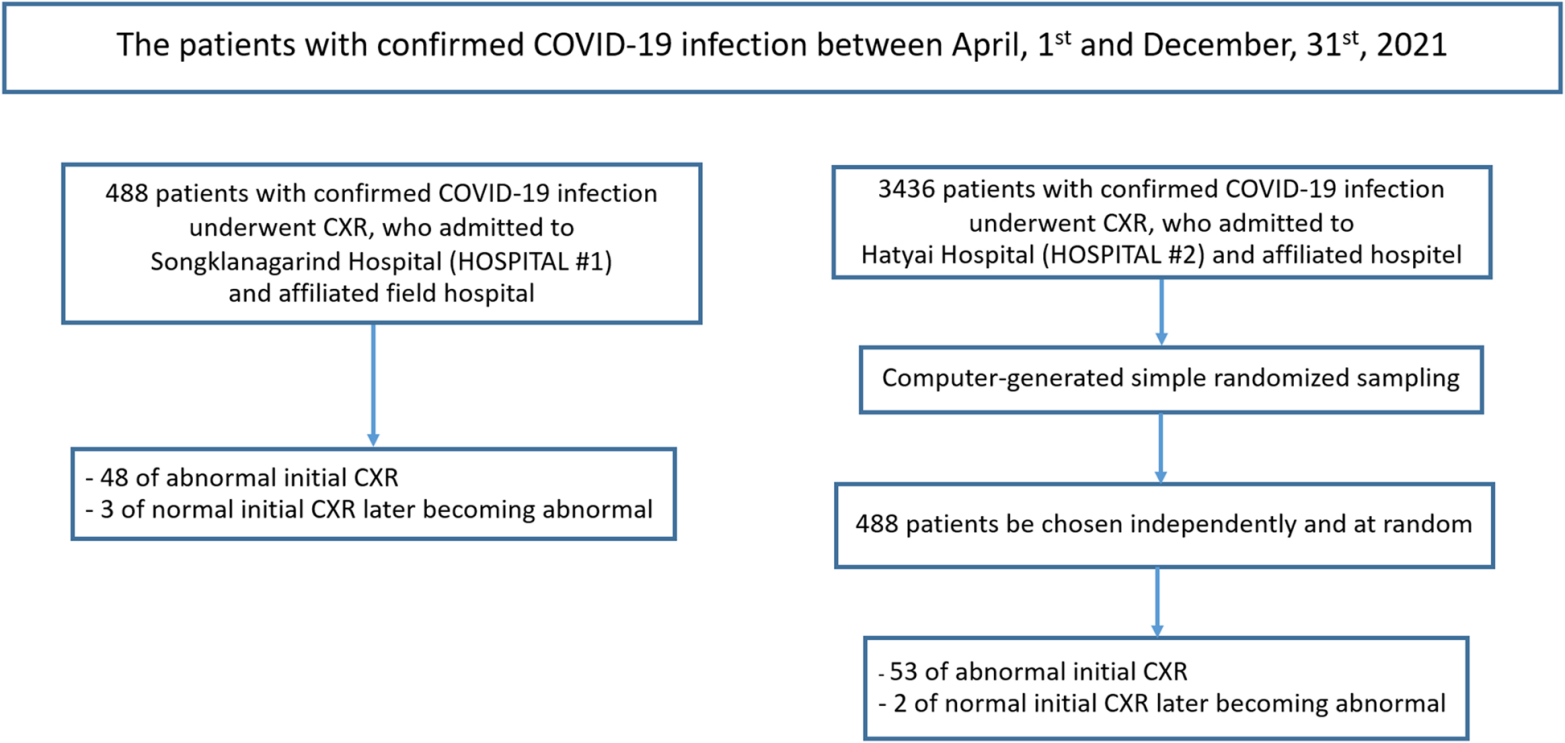
Flowchart of the Study. *COVID-19* Coronavirus disease-19, *CXR* Chest radiography

The sample size was calculated by using data from a previous study by Palabiyik et al.’s study [7]. The proportion of abnormal CXRs in pediatric patients with confirmed COVID-19 was 0.46. The margin of error was 0.1 (20% of the proportion). Thus, a minimum of 106 patients was require in this study. In conducting our research, one of our primary objectives was to achieve a balanced representation of patient samples from both HOSPITAL #1 and HOSPITAL #2 (Fig. 1). This balance is crucial for ensuring that our findings are representative and applicable across a broader patient population.

To accomplish this, we took a deliberate approach to patient enrollment. In HOSPITAL #1, we enrolled all patients, which amounted to a total of 488 individuals. This comprehensive approach allowed us to capture a complete picture of the patient population at HOSPITAL #1. For HOSPITAL #2, given the substantial patient registry of 3,436 individuals, we employed a simple random sampling method. This method involved using a computer program to randomly select 488 patients from the patient registry at HOSPITAL #2. We adopted this sampling technique to ensure that our sample from HOSPITAL #2 constituted a representative subset of the hospital's diverse patient population.

Finally, we recruited only patients who had abnormal initial CXRs (48 and 53 patients from HOSPITAL #1 and HOSPITAL #2, respectively) and those whose initial CXRs were normal but later became abnormal (3 and 2 patients from HOSPITAL #1 and HOSPITAL #2, respectively).

These pediatric patients presented with or without symptoms. All of them underwent CXR before or during admission. The initial CXR was performed approximately 0–2 days after a positive RT-PCR result. The CXR images were evaluated from our Picture Archiving and Communication Systems (PACS).

COVID-19 symptom severity was classified as asymptomatic infections; mild, upper respiratory tract infection or mild other symptoms (such as fever, fatigue, myalgia, sore throat, and running nose); moderate, pneumonia either with clinically or abnormal radiological findings; severe, pneumonia with desaturation or need for respiratory support; or critical, respiratory failure or life-threatening conditions [5].

### Imaging technique and analysis of chest radiographs

The posteroanterior (PA) or anteroposterior (AP) view of CXR was obtained using portable digital X-ray machines. The HOSPITAL #1 used Fuji FDR Nano (Fujifilm, Tokyo, Japan) and the scan parameters were 50–55 kV and 1.25–1.6 mAs for small children aged < 3 years as well as 75–100 kV and 2–4 mAs for children aged 3–18 years. The HOSPITAL #2 used Siemens MobileTT Elara Max (Siemens Healthcare, Erlangen, Germany) and the scan parameters were 50–55 kV and 1.25–1.6 mAs for small children aged < 3 years, 55–60 kV and 1.4–1.8 mAs for children aged 3–7 years, and 65–130 kV and 2.5–4 mAs for those aged > 7–18 years. In total, 125 of all 976 patients underwent CXR in a supine position, and the rest of them underwent CXR in an upright position. CXR follow-up examination was performed based on clinical criteria. Two radiologists, a fellowship-trained pediatric radiologist and a board-certified pediatric radiologist, retrospectively and independently reviewed all chest radiographs. If there was a discrepancy, another pediatric radiologist helped to reach a consensus interpretation. The radiographic patterns included lung abnormalities, the distribution of the lesion, and other findings (e.g., pleural effusion). We also categorized the radiographic features by age group: 0– < 5 years, 5– < 9 years, and 9–18 years.

**Fig. 2:**
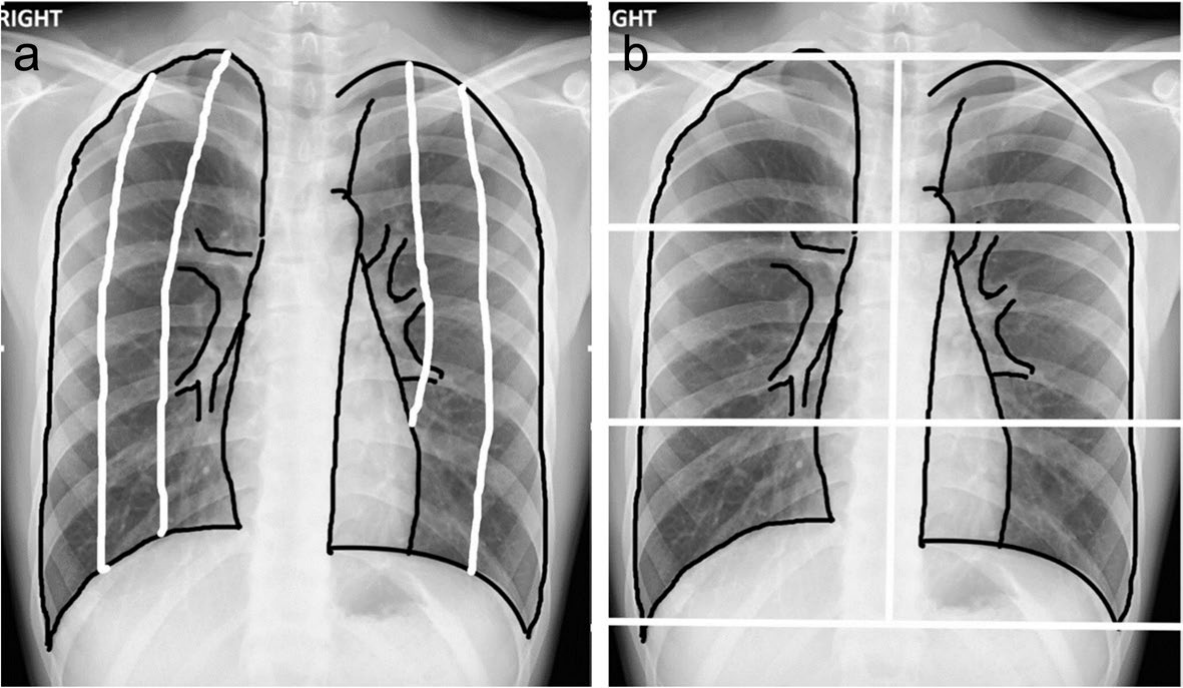
Distribution of lung zones. **a** The peripheral, middle, and perihilar lung zones are divided into three equal zones in each lung. **b** The upper zone extends from the apical lung to the superior portion of the hilum. The mid-zone is the space between the superior and inferior hilar regions. The lower zone extends from the inferior hilar margin to the costophrenic angle

The lung distribution was categorized as peripheral, perihilar predominance, or neither [17]. The lung zones were modified by dividing CXR into three fields for each lung (Fig. 2a, b) [18, 19]. The upper lung field extended from the apical lung to the superior portion of the hilum. The mid-lung field was the space between the superior and inferior hilar regions. The lower lung field extended from the inferior hilar margin to the costophrenic angle [20].

The structured reporting of CXR features for pediatric COVID-19 included a workup for pneumonia to classify the imaging findings into typical, indeterminate or atypical [2]. Typical findings were used to define the imaging pattern most suggestive of COVID-19 pneumonia, including bilateral peripheral and/or subpleural GGO and/or consolidation. An indeterminate finding was less specific than those of the typical group, e.g., unilateral non-segmental/lobar GGO or consolidation, bilateral peribronchial thickening and/or peribronchial opacity, or multifocal GGO/consolidation without any particular distribution. The definition of peribronchial thickening/ opacity is abnormal thickening of bronchial walls and detecting tram-track sign. Atypical findings included unilateral segmental/lobar consolidation, central distribution of parenchymal opacities, single round consolidation, pleural effusion, and lymphadenopathy. Such findings are infrequently observed in cases of COVID-19 pneumonia and suggest an alternative diagnosis.

### Radiographic scoring

To quantify CXR scoring for monitoring the severity of COVID-19 pneumonia, we used a modified version of a scoring system proposed by Wong et al. [4]. The total scores of 0–3 points were assigned to each lung depending on the extent of lung zone involvement (Fig. 2a). Lung zone involvement on CXR was classified as mild (score, 1–2 points), moderate (score, 3–4 points), and severe (score, 5–6 points) depending on number of lung zone involvement [20].

### Statistical analysis

The proportion of radiographic patterns of CXR was analyzed using Fisher’s exact test. Associations between categorical variables (sex, age, and CXR findings) and clinical outcomes (hospital stay, severity of disease, and viral load) were analyzed using the unpaired t-test. All results are presented with two-tails and a *p*-value of ≤ 0.05 was considered statistically significant. Statistical analyses were performed using the statistical program R (R Foundation for Statistical Computing, Vienna, Austria) by a statistician.

## Results

### Patient characteristics

Among the children diagnosed as having COVID-19 with abnormal chest radiographs, the demographic features of 106 cases are shown in Table 1 (59 boys [55.7%] and 47 girls [44.3%]; median age, 6 years [5, 13]; and age range, 18 days to 18 years). Forty-four of 106 children (41.5%) were asymptomatic. The clinical presentation by age revealed that most of the patients older than 9 years of age were symptomatic (28/62, 45.2%), whereas those in infant and toddler groups were asymptomatic (22/ 44, 50%). The mean duration of the onset from clinical symptom to date of performed CXR in symptomatic patients was 5.3 (1–14) days.

**Table 1:** Characteristics of the 106 children diagnosed with COVID-19 who underwent chest radiography

Characteristics	All (*N* = 106)	Asymptomatic (*n* = 44)	Symptomatic (*n* = 62)	*p*-value
**Sex**				0.425
Male, n (%)	59 (55.7%)	27 (61.4%)	32 (51.6%)	
Female, n (%)	47 (44.3%)	17 (38.6%)	30 (48.4%)	
**Age, median [IQR**]	6 [4, 12]	5 [3, 8]	8 [4, 14]	0.006*
**Age group, n (%)**				0.022*
0– < 5 years	40 (37.7%)	22 (50%)	18 (29%)	
5– < 9 years	29 (27.4%)	13 (29.5%)	16 (25.8%)	
9–18 years	37 (34.9%)	9 (20.5%)	28 (45.2%)	
**Age < 1 year**	7 (6.6%)	3 (6.8%)	4 (6.5%)	1.000
**Cycle threshold of RT-PCR SARS-**				
**CoV-2** (*n* = 22)^a^				
*ORF*-gene, mean (SD)	22.6 (6.1)	22.1 (5.3)	23.2 (7)	0.682
N-gene, mean (SD)	21.1 (5.9)	20.5 (6.6)	21.8 (5.3)	0.619
**Treatment**				
Required oxygen support, n (%)	5 (4.7%)	2 (4.5%)	3 (4.8%)	1.000
Favipiravir, n (%)	34 (32.1%)	7 (15.9%)	27 (43.5%)	0.005*
Remdesivir, n (%)	9 (8.5%)	4 (9.1%)	5 (8.1%)	1.000
Antibiotic, n (%)	9 (8.5%)	4 (9.1%)	5 (8.1%)	1.000
Steroid, n (%)	10 (9.4%)	4 (9.1%)	6 (9.7%)	1.000
**CXR results**				0.647
Abnormal initial CXR findings, n (%)	101 (95.3%)	41 (93.2%)	60 (96.8%)	
Normal initial CXR findings, n (%)	5 (4.7%)	3 (6.8%)	2 (3.2%)	
**Severity score of abnormal CXR**				0.737
**Images**				
Mild (score, 1–2), n (%)	81 (76.4%)	32 (72.7%)	49 (79%)	
Moderate (score, 3–4), n (%)	21 (19.8%)	10 (22.7%)	11 (17.7%)	
Severe (score, 5–6), n (%)	4 (3.8%)	2 (4.6%)	2 (3.3%)	

*COVID-19* Coronavirus disease-19, *CXR* Chest radiography, *IQR* Interquartile range, *N/A* Not applicable, *ORF* Open reading frame, *RT-PCR* Reverse transcriptase-polymerase chain reaction, *SARS-CoV-2* Severe acute respiratory syndrome coronavirus 2, *SD* Standard deviation

^*^Statistically significant, *p*-value ≤ 0.05

^a^Large proportion of missing data in the cycle threshold of RT-PCR SARS-CoV-2 (84/106 cases)

Three infants and two children in early childhood had severe pneumonia that required respiratory support; noninvasive respiratory support was provided in four cases and endotracheal intubation was provided in one case. Two symptomatic infants younger than 1 year of age had abnormal CXR findings at initial presentation. One infant with a contact history of maternal COVID-19 and one asymptomatic child had normal initial CXR findings that later became abnormal. Then, oxygen desaturation occurred during admission. A 22-month-old boy had severe COVID-19 pneumonia with respiratory failure at initial presentation. His chest radiograph showed bilateral multifocal patchy opacities and peribronchial thickenings. Four of five cases (except an asymptomatic child) received favipiravir, remdesivir, antibiotic, and a steroid. Their length of hospital stay (LOHS) was approximately 10–14 days. There was no case of mortality in our study.

### Radiographic patterns and CXR reporting

Abnormal CXR findings occurred in 106 of 976 cases (10.9%). Fifty-one and 55 cases were from HOSPITAL #1 and HOSPITAL #2, respectively. Overall, 101 of 106 cases (95.3%) (48 and 53 cases from HOSPITAL #1 and HOSPITAL #2, respectively) had abnormal initial CXR findings, and only five of 106 cases (4.7%) (three and two cases from HOSPITAL #1 and HOSPITAL #2*, respectively) had normal initial chest radiographs, although they became abnormal later.

The lower lung zone (56/106, 52.8%) was the most common area affected, and most children had bilateral or right lung involvement. Additionally, 31.1% and 21.7% of the lesions occurred in the right lower lobe and both lower lobes, respectively. An indeterminate chest imaging pattern was commonly found in children with COVID-19 pneumonia (92/106, 86.8%). The most common radiographic finding was peribronchial thickening (54/106, 51%). The distribution of lung abnormalities on chest radiographs and CXR reporting are presented in Table 2.

**Table 2:** Distribution of abnormalities on CXR and CXR reporting

Distribution of abnormal CXR findings	Total = 106
Right lung	46 (43.4%)
Left lung	16 (15.1%)
Both lungs	44 (41.5%)
Upper lung field	2 (1.9%)
Lower lung field	56 (52.8%)
No zonal predominant	48 (45.3%)
**CXR reporting**	
Typical	13 (12.3%)
Indeterminate	92 (86.8%)
Atypical	1 (0.9%)
**Site of involvement of initial CXR findings in 106 cases**	
**Affected lung lobe**	**No. of cases (n, %)**
Right lower lobe	33 (31.1%)
Both lower lobes	23 (21.7%)
Left lower lobe	7 (6.6%)
Both middle and lower lobes (except both upper lobes)	6 (5.6%)
Right middle and lower lobes	6 (5.6%)
Right middle lobe	4 (3.8%)
Right lower lobe and lingular segment	4 (3.8%)
Left lower lobe including the lingular segment	4 (3.8%)
Lingular segment	4 (3.8%)
Diffuse, both lungs	2 (1.9%)
Others	13 (12.3%)

*CXR* Chest radiography, *no.* Number

### Radiographic scoring

Regarding the severity of COVID-19 pneumonia based on abnormal CXR findings, 81 of 106 cases (76.4%) had mild lung abnormalities. More extensive lung involvement was found in 21 (19.8%) and 4 (3.8%) cases of the pediatric patients who had moderate and severe lung abnormalities, respectively. No significant differences were observed between total lung scores and the asymptomatic and symptomatic groups (Table 1). There was no statistical significance in the distribution of severity scores among different pediatric age groups (Table 3).

**Table 3:** Radiographic features by age group

Variable (n, %)	0– < 5 years *N* = 40	5– < 9 years *N* = 29	9–18 years *N* = 37	Total *N* = 106	*p*-value
**Density category**					
Ground-glass opacity	3 (7.5%)	4 (13.8%)	19 (51.4%)	26 (24.5%)	< 0.001*
Patchy opacity	1 (2.5%)	1 (3.5%)	2 (5.4%)	4 (3.8%)	0.832
Peribronchial thickening	25 (62.5%)	19 (65.5%)	10 (27%)	54 (51%)	0.001*
Reticulonodular infiltration	2 (5%)	1 (3.4%)	0 (0%)	3 (2.8%)	0.49
Mixed	9 (22.5%)	4 (13.8%)	6 (16.2%)	19 (17.9%)	0.613
**Lung involvement**					
Peripheral zone	0 (0%)	2 (6.9%)	9 (24.3%)	11 (10.4%)	< 0.001*
Perihilar zone	6 (15%)	3 (10.3%)	2 (5.4%)	11 (10.4%)	0.478
Neither peripheral nor perihilar	34 (85%)	24 (82.8%)	26 (70.3%)	84 (79.2%)	0.242
**CXR reporting (*N* = 106)**					
Typical	5 (12.5%)	1 (3.4%)	7 (18.9%)	13 (12.3%)	0.164
Indeterminate	35 (87.5%)	27 (93.2%)	30 (81.1%)	92 (86.8%)	0.393
Atypical	0 (0%)	1 (3.4%)	0 (0%)	1 (0.9%)	0.274
**Severity score of abnormal CXR images**					0.231
Mild (score, 1–2), n (%)	28 (70%)	23 (79.3%)	30 (81.1%)	81 (76.4%)	
Moderate (score, 3–4), n (%)	8 (20%)	6 (20.7%)	7 (18.9%)	21 (19.8%)	
Severe (score, 5–6), n (%)	4 (10%)	0 (0%)	0 (0%)	4 (3.8%)	

*CXR* Chest radiography

^*^ Statistical significance *p*-value ≤ 0.05

### Radiographic features by age groups

**Fig. 3:**
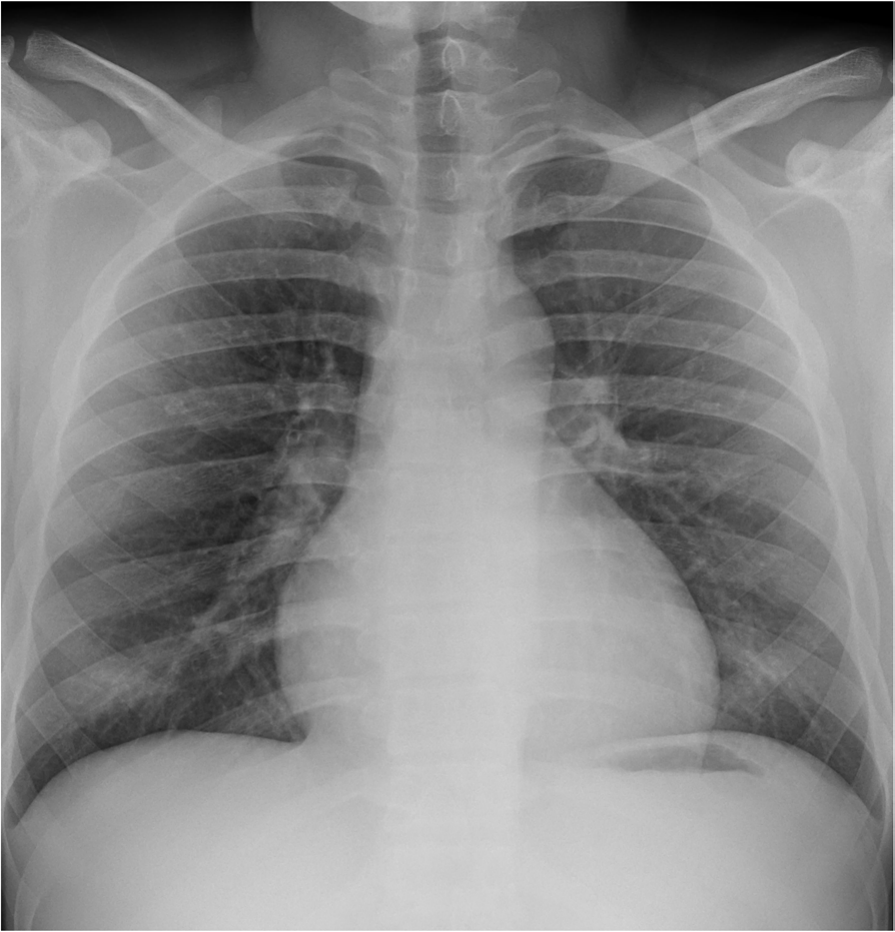
A 17-year-old boy with COVID-19 pneumonia who presented with an upper respiratory tract infection. The chest radiograph shows bilateral ground-glass opacities in both lower lung zones. The findings are consistent with the typical multifocal pattern of COVID-19 pneumonia. The severity scoring in this patient was calculated as the sum of the right lung scores and the left lung scores (total scores = 2 + 2 = 4). COVID-19, coronavirus disease-19

**Fig. 4:**
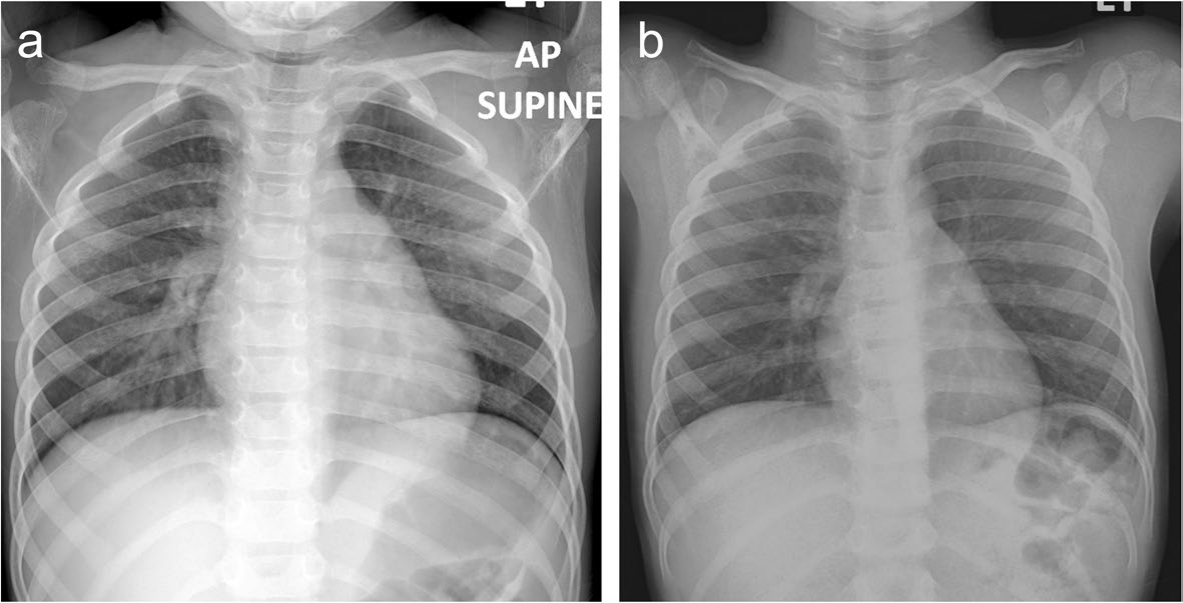
A 4-year-old girl with COVID-19 pneumonia who presented with a low-grade fever. **a** The initial chest radiograph shows bilateral peribronchial thickening, predominantly in the right lower lung field. The findings are consistent with the indeterminate pattern of COVID-19 pneumonia. The severity scoring in this patient was calculated as the sum of the right lung scores and the left lung scores (total scores = 2 + 1 = 3). **b** On day 6 of admission, the radiographic evaluation reveals an almost complete resolution of the infiltrative lung involvement. The follow-up severity score in this patient was calculated as the sum of the right lung scores and the left lung scores (total scores = 1 + 0 = 1). COVID-19, coronavirus disease-19

GGOs were commonly observed in children aged > 9 years (19/26, 73.1%) (Fig. 3), whereas, peribronchial thickening was predominantly found in newborns, infants, and toddlers (25/54, 46.3%) (Fig. 4a, b). Patchy opacity was a less common finding. The distribution of these lesions was neither peripheral nor perihilar (84/106, 79.2%) (Table 3).

### Severity scoring of CXR in pediatric patients with COVID-19

There were significant differences between severity lung scores and medical treatments; three of four children (75%) who had severe lung abnormalities received favipiravir, remdesivir, an antibiotic, and a steroid. Favipiravir was used more frequently than other drugs in all age groups (34/106, 32.1%) (Table 4).

**Table 4:** Severity scoring of CXR in pediatric patients with COVID-19

**Variable (n, %)**	**Total** ***N*** **= 106**	**Severity score of abnormal CXR findings**			***p*-value**
		**Mild (*n* = 81)**	**Moderate (*n* = 21)**	**Severe (*n* = 4)**	
**Treatment**					
Favipiravir, n (%)	34 (32.1%)	24 (29.6%)	7 (33.3%)	3 (75%)	0.177
Antibiotic, n (%)	9 (8.5%)	3 (3.7%)	3 (14.3%)	3 (75%)	< 0.001*
Steroid, n (%)	10 (9.4%)	4 (4.9%)	3 (14.3%)	3 (75%)	< 0.001*
Remdesivir, n (%)	9 (8.5%)	3(3.7%)	3 (14.3%)	3 (75%)	< 0.001*
Required oxygen support, n (%)	5 (4.7%)	1 (1.2%)	2 (9.5%)	2 (50%)	0.002*
Cycle threshold of RT-PCR SARS-					
CoV-2 (*n* = 22)^a^					N/A
*ORF*-gene, mean (SD)	22.6 (6.1)	23.4 (6.7)	22.4 (5.3)	18.8 (5.1)	
*N*-gene, mean (SD)	21.1 (5.9)	20.8 (6.7)	23.1 (4.3)	16.8 (2.4)	
**Follow-up CXR (n, %)**	**Total** ***N*** **= 40**	**Severity score of abnormal CXR findings**			***p*-value**
		**Mild (*n*** **= 28)**	**Moderate (*n*** **= 9)**	**Severe (*n*** **= 3)**	
Resolution	25(62.5%)	17 (60.7%)	6 (66.7%)	2 (66.7%)	1.000
Progression	6 (15.4%)	4 (14.3%)	1 (11.1%)	1 (33.3%)	0.387
Stable	4 (10.3%)	3 (10.7%)	1 (11.1%)	0 (0%)	1.000
Normal initial CXR findings later became abnormal	5 (12.8%)	4 (14.3%)	1 (11.1%)	0 (0%)	1.000

*CXR* Chest radiography, *COVID-19* Coronavirus disease-19, *N/A* Not applicable, *RT-PCR,* Reverse transcriptase-polymerase chain reaction, *ORF* Open reading frame, *SARS-CoV-2* Severe acute respiratory syndrome coronavirus 2, *SD* Standard deviation

^*^Statistical significance *p*-value ≤ 0.05.

^a^Large proportion of missing data in the cycle threshold of RT-PCR SARS-CoV-2 (84 of 106 cases)

Only six cases (15.4%) had progressive lung involvement during admission. Most children (25/40, 62.5%) had resolution of the previous lung abnormalities at the time of follow-up. Only six patients had progressive lung involvement at the time of follow-up.

Based on government policy, the LOHS was no more than 10 days. In case of clinical symptom was severe or did not improve, the LOHS could extend beyond 10 days. The maximum LOHS in this study was 20 days.

## Discussion

In Thailand, the third outbreak of COVID-19 occurred in April 2021, coinciding with an increasing proportion of the Delta variant, which is more transmissible and might cause more severe illness than other virus strains during that period. We assumed that most of the patients with COVID-19 in our study were infected with the Delta variant. The proportion of pediatric patients with COVID-19 in our study, who had abnormal radiographic features (106/976, 10.9%), was lower than that in a previously published study (27/59, 46%) [7]. It could be attributed to the different COVID-19 variant between Palabiyik et al.’s study and our study. Our outcomes suggest that an initial CXR can have a role in the initial screening if there is high clinical suspicion of COVID-19 pneumonia. Therefore, our institutions did not routinely perform follow-up chest radiographs in all pediatric patients with COVID-19 who had initially normal CXR findings at presentation. Abnormal CXR features often progress over time, as correlated with clinical information [21]. Overall, 3.4% of adult patients with COVID-19 pneumonia developed ARDS and had a slow resolution of abnormalities over weeks [22]. However, children seem to have milder symptoms and lower mortality and 2.1% of the cases in the pediatric group (younger than 18 years of age) showed progression to severe forms of disease [23, 24]. Herein, only five of 106 pediatric cases (4.7%) had normal initial CXR findings that later became abnormal within 5 days on average. Accordingly, follow-up CXR is necessary for pediatric patients with clinical deterioration or complicated pneumonia after treatment.

Pediatric patients, particularly those aged > 9 years, were symptomatic. However, the children aged < 5 years were asymptomatic. A few studies hypothesized that the younger children might have protective immune system and healthier respiratory endothelial function than older children and adult [25, 26]. We hypothesized that older children and adolescents can communicate their illness more than infants and those in early childhood.

Prior research has used combined CXR and chest CT (sample size = 80) and chest CT alone (varying sample sizes from 1 to 171 cases) to identify the imaging characteristics of pediatric COVID-19 pneumonia [27–30]. Although chest CT provides more details in mediastinal or hilar adenopathies, more accuracy in lobar distribution, and more sensitivity to detect GGO than CXR, there are still inconsistent CT descriptors in pediatric COVID-19 pneumonia [30]. Moreover, extra information from the initial chest CT scan did not alter the course of treatment for the patient, and the scan should only be used for severely symptomatic patients and/or pre-existing comorbidities [10, 11]. The necessity of chest CT and the radiation risk need to be balanced. The previous study used CT for imaging CT features in COVID-19 pneumonia with severe-to-critical cases (57.5%) and comorbid conditions (50%), whereas our study had only 4.7% (5/106) with severe pneumonia and appropriately focused on CXR [27].

The main finding of peribronchial thickening on CXR in pediatric patients with COVID-19 pneumonia was consistent with that of a previously published article [13]. Peribronchial thickening was the most common finding in our study followed by GGO. The distribution of these lesions was neither peripheral nor perihilar. The combination of peripheral and perihilar zones was found most frequently in all age groups, which is in concordance with the result of another study [7]. Peripheral distribution in children may not be as common as that in adults [31].

In our study, 31.1% and 21.7% of the lesions occurred in the right lower lobe and both lower lobes, respectively. Similar to another study, unilateral lung involvement was more frequent in children than in adults (30% versus 11.7%) [32]. Research conducted on children with COVID-19 pneumonia using chest CT findings assessment also revealed that unilateral lung involvement was greater than bilateral lung involvement (29.4% versus 24.7% in Qi et al.’s study, 54.5% versus 45.5% in Zhang et al.’s study, and 55% versus 45% in Katal et al.’s study) [28–30]. Distribution was slightly predominant in the right lower lung in our study, which was different from that in a previous study, in which 59–73% of the lesions were in the left lower lung zone [7, 29]. Subtle lesions (especially peribronchial thickening) on CXR may be obscured by cardiac shadow, which mostly occupies the left lung.

Only a few studies have categorized the imaging features into age groups [33, 34]. Our study found the significant differences between age groups and density categories. GGO was predominant in school-age children and those aged > 9 years (73.1%). Whereas peribronchial thickening was the most common in children aged < 5 years (46.3%). These findings are similar to those of a recent study in which GGO/consolidations were more prevalent in older children and perihilar markings were more prevalent in younger children, although there was some controversy regarding age [33]. Nino et al.’s study found that GGO/consolidation was common in aged 3–10 years (36.4%) and 11–19 years (42.4%), whereas peribronchial markings were common in aged 0–2 years (37.5%) and 3–10 years (40.6%) [33]. However, there was no significant difference regarding GGO and peribronchial thickening by age groups in Bayramoglu et al.’s study [34]. The possible explanation could be the small sample size of the presence of GGO (2/20 patients aged 0– < 6 years and 3/26 patients aged 12–18 years) and peribronchial thickening (6/20 patients aged 0– < 6 years and 1/26 patients aged 12–18 years) on the chest radiograph in that study [34].

The study's small sample size of GGO (5/69 patients) and the presence of peribronchial thickening (7/69 patients) on the chest radiograph could be the cause.

The distribution of radiographic severity scores across various pediatric age groups was initially determined by our study. We discovered that 76.4% of patients across all age categories had a mild severity score. There was no statistical significance, despite the fact that the patients with severe radiographic score were limited to the age group < 5 years (Table 3). Our study also analyzed the radiographic severity scoring (in initial and follow-up CXR), correlation with clinical context and medical treatments (Tables 1 and 4). Most pediatric patients with COVID-19 pneumonia in our study had mild lung abnormalities. More extensive lung involvement was found in 21 (19.8%) and 4 (3.8%) pediatric patients with moderate and severe lung abnormalities, respectively. No significant differences were observed between total lung scores and symptoms in the initial CXR. The correlation between initial radiological severity scoring and clinical outcomes has been studied in earlier research [27, 33]. The high severity score was associated with hospitalization, the need for ICU admission, oxygen supplementation, and COVID-19 complications [27, 33]. Similarly, our study found significant differences between severity of lung score and oxygen requirement and additional details in medical treatments. The severe lung scoring group received favipiravir, remdesivir, an antibiotic, and a steroid more than the mild and moderate lung scoring groups.

In our subgroup with radiographic recovery after treatment (25/40, 62.5%), there was no statistically significant difference between their median LOHS and recovery of approximately 10 days. In a subgroup analysis of 40 cases, six patients (15%) had progressive lung involvement at the time of follow-up. However, there was no significant difference between the radiographic findings in follow-up CXR and radiographic severity scores. Unfavorable outcomes were more frequent when initial CXR showed bilateral involvement and a combination of patchy opacities and peribronchial thickenings [12]. We still believe that chest imaging should be used as an adjunct tool in the monitoring of the severe course of COVID-19 pneumonia until further evidence is obtained.

Our study has a few limitations. First, all pediatric patients with COVID-19 were not followed until complete clearance of the radiographic abnormalities. Second, some of the clinical data collected were limited because of incompleteness of the electronic medical records. Third, the time intervals between symptom onset and a sequential chest radiograph were not uniform, affecting the accuracy of our analysis. Finally, the subtle radiographic abnormalities may limit reliability in suboptimal assessment and are highly variable depending on the grade of inspiration on the chest radiographs. Future studies are needed to develop a clinical practice guideline for follow-up CXR to improve the monitoring of the disease course and the time of resolution.

## Conclusions

Our study demonstrated significant differences between age groups and density categories in the pediatric patients with COVID-19 pneumonia. The most common radiographic feature in infants and those in early childhood aged < 5 years was peribronchial thickening; however, GGO was more frequent in children aged > 9 years. Both lower lobes were predominantly affected, but the right lower lung was slightly more affected than left lower lung. Chest radiographs are usually indicated to establish a baseline study and to assess for an alternative diagnosis. The role of total severity lung scores in the initial CXR of COVID-19 pneumonia in pediatric patients might predict the clinical outcome and medical treatments.

## Data Availability

The datasets analyzed in this study are available from the corresponding author upon the reasonable request.

## Abbreviations

*AP*: Anteroposterior
*ARDS*: Acute respiratory distress syndrome
*COVID*: Coronavirus disease
*Ct*: Cycle threshold
*CXR*: Chest radiography
*GGO*: Ground-glass opacity
*LOHS*: Length of hospital stay
*PA*: Posteroanterior
*PACS*: Picture Archiving and Communication Systems
*RT-PCR*: Reverse transcriptase-polymerase chain reaction
*SARS-CoV-2*: Severe acute respiratory syndrome-corona virus-2
